# Supplementary material for: Are carbon emissions trading and green financial instruments synergistic? -Comprehensive quantitative research based on content analysis
Source: PLoS One. 2024 Mar 7;19(3):e0298601. doi: 10.1371/journal.pone.0298601 (PMC10919601; doi:10.1371/journal.pone.0298601)
Supplement: S1 Data — (ZIP) [file pone.0298601.s001.zip › Data/地级市Z_F工作报告.zip!/╕ú╜¿/╕ú╓▌╩╨/└┤╫╘╣1⁄2╓┌║┼í╛┬φ┐╦╩2╛▌═°í┐_2014.docx]

各位代表：

　　现在，我代表福州市人民政府向大会作政府工作报告，请各位代表审议，并请市政协各位委员和其他列席人员提出意见。

　　一、2013年工作回顾

　　2013年，在省委、省政府和市委的正确领导下，我市各级政府认真贯彻落实党的十八大、十八届三中全会和习近平总书记系列重要讲话精神，统筹稳增长、调结构、促改革、惠民生，较好地完成了市十四届人大二次会议确定的任务。

　　（一）经济发展稳中有进、稳中向好。预计2013年全市生产总值4700亿元，增长11.5%；公共财政总收入689.12亿元，增长15.4%，其中，地方公共财政收入453.97亿元，增长18.8%；固定资产投资3832.53亿元，增长18.5%；外贸出口192亿美元，比上年实际数（剔除政策性因素)增长6%；按验资口径实际利用外资14.06亿美元，增长5%；社会消费品零售总额2611亿元，增长15.6%；城镇居民人均可支配收入32130元，增长9.3%；农民人均纯收入12870元，增长12%。“五大战役”完成投资1900亿元，为年度计划的134.6%；实施市级重点项目300项，完成投资1170亿元，为年度计划的125.5%。福清、闽侯、长乐、连江被评为全省县域经济实力十强县，闽侯、连江被评为全省经济发展十佳县。

　　（二）产业结构调整和转型升级步伐加快。全市新增1个千亿产业集群和2家百亿企业，规模以上工业增加值1653亿元，增长13.5%。产业大项目加快向南北“两翼”地区集聚，有66个项目动工建设、77个项目建成投产。新兴产业较快发展，高新技术产业产值突破2800亿元，福州高新区海西园一期基本建成，闽台（福州)蓝色经济产业园、临空经济区等园区起步建设。自主创新能力不断增强，福州蝉联全国科技进步先进市称号，新增2家国家地方联合工程研究中心、37家院士（专家)工作站、2家博士后科研工作站。中国福州海西引智试验区建设扎实推进，福州人才市场挂牌成立，福州与清华大学、中国人民大学等8所高校签订战略合作协议。金融、现代物流、电子商务、服务外包、旅游、文化创意、会展、总部经济等服务业快速发展，普洛斯物流、华侨城旅游等一批大项目落户福州，苏宁广场、五四北泰禾广场等城市综合体建成开业，海峡股权交易中心、通用航空产业基地等投入运营。

　　（三）改革开放不断深化拓展。构建形成以“四大投”与三个专业公司为构架的国资管理运作新体制。道路清扫保洁、园林绿化养护等领域市场化运作全面开展。行政审批制度改革不断深化，市行政服务中心成为国家级服务业标准化试点单位，审批服务当场办结率78%，群众满意率99.99%，市民服务中心建设进展顺利。福州高新区成功实行新管理体制。事业单位分类改革、公立医院改革、义务教育“小片区”管理改革、市属文化单位改制等取得新的成果。农村综合改革持续深化，农村集体土地所有权确权登记、林权配套制度改革等有序推进。开放型经济加快发展，签约对接“三维”项目292项，意向总投资2693亿元；新批千万美元以上外（台)资项目47项。江阴港区汽车整车进口口岸通过国家验收，黄岐对台小额贸易口岸获批试行更开放式管理。成功举办“5·18”海交会和海峡青年节、两岸船政文化研讨会、海峡两岸合唱节等对台特色交流活动，“三坊七巷”被确定为海峡两岸交流基地。积极支持平潭开放开发，渔平高速公路延伸线基本建成，福平铁路动工建设，闽江北水南调（平潭引水)工程等项目扎实推进。福莆宁同城化步伐加快。对口援建工作有效开展，榕港榕澳合作和侨务、外事、异地商会等工作取得新成果，福州与匈牙利塞格德市、俄罗斯鄂姆斯克市建立友好交流城市关系。

　　（四）城市面貌发生较大改观。编制完成历史文化名城保护、地下空间、旧城区步行慢道系统等专项规划，基本实现中心城区控规全覆盖。重点区域开发建设步伐加快，螺洲大桥、琅岐闽江大桥等一批项目建成或基本建成，南江滨东段二期等一批项目启动建设。对外通道加快拓展，向莆铁路、福永高速公路建成通车，实现所有县（市)通达高速公路。县（市)城关改造提升成效明显，13个省、市试点小城镇建设扎实推进。中心城区环境综合整治全面推进，完成57条街巷整治、400多幢建筑立面改造和26座高架桥、人行天桥绿化花化，建成11个城市公园和50.5公里城市绿道与慢行系统。实施旧屋区改造440万平方米，综合整治老旧住宅小区268个。新改扩建城市道路20条。新增更新公交车779辆、出租车763辆，新辟和优化公交线路69条，福州成为国家公交都市建设示范工程试点城市。地铁1号线建设和地铁2号线前期工作全面推进。综合治理23条内河，闽江经光明港至八一水库步行道全线贯通，内河整治工程荣获中国人居环境范例奖。违法建设、非法采砂、渣土车违规等专项整治扎实推进。“数字城管”系统一期、智能交通控制中心三期等建成投入使用。

　　（五）城市环境质量位居全国前列。城市空气质量优良率94%，综合指数排名持续位居全国大中城市前列。实施82项重点节能项目和335项重点减排项目，一批环保项目建成投入使用，预计可完成省下达的年度节能减排任务。“四绿”工程建设成效明显，新增城市绿地567万平方米，造林绿化39.15万亩，森林覆盖率55.3%，人均拥有公园绿地面积12.8平方米。重点流域水环境持续改善，闽江河口湿地获评“中国十大魅力湿地”。完成水土流失治理14.94万亩。马尾、福清、长乐、永泰等通过国家级生态县（市)区评估，新创建52个国家级生态乡镇、179个市级生态村。

　　（六）“三农”工作稳步推进。粮食产量保持稳定，农林牧渔业总产值690亿元，增长4.7%。市级农业产业化龙头企业年销售额突破500亿元，中以示范农场等国际合作项目落户福州。福州获得“中国金鱼之都”、“中国鳗鲡之都”称号，福州茉莉花种植与茶文化系统入选首批中国重要农业文化遗产。实施葫芦门水库等21个重大水利项目建设，新建改造农村公路255公里。扶贫攻坚和县（市)区对口协作力度加大，“百村竞赛”和“美丽乡村”建设活动深入开展，新建农村户用沼气池1000口，新建改建农村无害化卫生户厕3922户，新建空白村卫生所89个，解决84.3万农村人口饮水安全问题，实施农村造福工程危房改造1750户。

　　（七）各项社会事业协调发展。各级各类教育加快发展，新建、改扩建公办幼儿园45所，扩容中小学学位1.02万个，福州八中三江口校区等重大项目建成投入使用，全面完成义务教育标准化学校创建任务。医疗卫生服务体系逐步完善，新农合大病补偿标准、基本公共卫生服务人均经费标准进一步提高，新增医疗机构床位715张，组建成立全省首个医疗联合体。市工人文化宫建成投入使用。朱紫坊、上下杭、烟台山等历史文化街区（风貌区)保护修复工作启动实施，闽都民俗园等项目建成开业。新福州人歌手大赛、福州小茉莉合唱团以及闽剧《红裙记》、《林则徐复出》等获得国家级大奖。寿山石文化节、文化艺术周、闽都文化论坛、环福州·永泰国际公路自行车赛、中华龙舟大赛等大型文化体育活动成功举办。首届全国青年运动会各项筹备工作有序推进。低生育水平保持稳定，人口自然增长率控制在7‰以内。第三次全国经济普查顺利开展。民族团结进步，宗教和谐稳定。新闻出版和广电事业协调发展，妇女、儿童、老龄、残疾人、慈善等工作得到加强，国防动员、双拥共建、民兵预备役、海防、人防等工作取得新的进步。

　　（八）人民生活水平持续提高。全市各级财政用于民生支出396.31亿元，占公共财政支出的74.3%。年初确定的25件63项为民办实事项目如期完成。城镇新增就业14.5万人，转移农业富余劳动力5.3万人，城镇登记失业率控制在2.5%以内。在全省率先实施城镇居民大病保险，居民社会养老保险、最低工资标准实现城乡统一，城乡低保标准、农村五保供养标准进一步提高。市场物价基本稳定，居民消费价格总水平上涨2.7%。主要食品安全检测指标总体达标。基本建成保障性住房18549套，超额完成省下达的任务。“平安福州”建设扎实推进，人民群众对社会治安满意率达95.06%，安全生产事故发生起数和死亡人数分别下降9.7%、14.9%。有效抗击“苏力”等台风，最大限度减少了人民群众生命财产损失。信访、行政复议、人民调解、法律援助、社区矫正等工作进一步加强。

　　（九）政府工作作风切实改进。认真执行中央“八项规定”，全市性会议、文件简报数量和“三公”经费支出分别下降20%、28%和13%。深入开展“四下基层”、“四个万家”等活动，全市处级以上领导干部深入基层、企业约9500人次，解决实际问题约1.2万个。大力弘扬“马上就办”优良传统，效能建设、绩效管理不断加强，民主评议政风行风等活动受到群众欢迎与好评。政府信息公开、“中国福州”门户网站建设等工作走在全国前列。惩治和预防腐败体系进一步完善，行政监察、审计监督等工作力度持续加大。认真执行人大及其常委会决定决议，自觉接受人大及其常委会的法律监督、工作监督和政协的民主监督。提请市人大常委会审议地方性法规草案2件，制定政府规章和规范性文件27件。办复532件省、市人大代表建议、批评、意见和622件省、市政协提案，满意率分别为98%和99%。

　　各位代表，过去一年的成绩来之不易，这是在省委、省政府和市委的正确领导下，全市人民团结奋斗的结果。在此，我代表市人民政府，向全市人民致以崇高的敬意！向人大代表、政协委员、各民主党派、工商联、各人民团体、无党派人士、离退休老同志和社会各界人士，向中直、省直机关企事业单位和驻榕部队、武警官兵、公安政法干警，向关心支持福州发展的港澳台同胞、海外侨胞和国际友人，表示衷心的感谢！

　　在看到成绩的同时，我们也必须正视当前福州发展中存在的困难和问题，主要是：产业集群发展水平不高，龙头企业和知名品牌较少，自主创新能力较弱；城乡规划建设管理水平还需提高，村镇规划滞后、执行不力，环境综合整治、旧屋区改造等任务比较重，群众对交通拥堵、违法建设等问题反映比较强烈；节能减排攻坚压力较大，环境保护和生态建设任务仍然艰巨；民生保障水平有待提升，优质教育、医疗、文化等资源总量不足、分布也不够均衡，住房、就医、就学、食品药品安全等方面需要解决的问题仍比较多；社会服务管理需要加强，维护和谐稳定的压力还比较大；一些干部责任感、事业心不够强，“庸懒散”和消极腐败等现象仍然存在。对此，我们将切实采取有效措施，认真加以解决。

　　二、2014年工作安排

　　各位代表，2014年是全面贯彻落实党的十八届三中全会精神、全面深化改革的第一年，也是认真实施“十二五”规划、力争率先全面建成小康社会的关键一年。福州作为改革开放先行城市，改革开放始终是我们必须牢牢把握的“生命线”。面对新形势、新任务，我们要把改革创新贯穿于经济社会发展各个领域各个环节，全力推进福州新区开放开发，在更高起点上加快建设闽江口金三角经济圈，促进经济持续健康发展、社会和谐稳定。

　　今年全市经济社会发展主要预期目标是：地区生产总值增长11%左右；地方公共财政收入增长12%；固定资产投资增长18%；外贸出口增长7%；按验资口径实际利用外资增长5%；社会消费品零售总额增长15%；居民消费价格总水平涨幅控制在3.5%左右；城镇居民人均可支配收入增长10%，农民人均纯收入增长11%；城镇登记失业率控制在3.5%以内；人口自然增长率控制在9.3‰以内；完成单位生产总值能耗、化学需氧量、二氧化硫、氨氮、氮氧化物等年度节能减排任务。

　　为实现上述预期目标，重点抓好以下八个方面工作：

　　（一）奋力开创改革开放新局面。

　　全力突破重点领域和关键环节改革。加强国有资产监管，推动市属国有企业加快整合重组和完善现代企业制度，推进市直部门与所属企业及经营性资产全面脱钩。进一步鼓励发展非公有制经济和混合所有制经济，鼓励非公资本投资城市道路、广场、地下管网、公共停车场等市政工程和学校、医院、养老院等社会公益项目，推动非公资本参与城镇供水、燃气和污水垃圾无害化处理等建设。深化投融资体制改革，着手设立市属金控集团和创投公司，抓好海峡环保等市属企业上市工作。推行商事登记制度改革，最大限度降低企业准入门槛和创业成本。加快建设规范统一的公共资源交易服务平台。深化土地使用制度改革，完善农村征地补偿标准和多元补偿机制，稳妥推进农民住房财产权抵押、担保、转让试点。深化财税体制改革，建立健全事权与支出责任相适应的财政制度，推行政府全口径预算管理，从严控制“三公”经费等一般性支出。

　　全面实施大开放战略。积极创造条件力争福州新区上升为国家发展战略。主动争取平潭综合实验区政策辐射，着力在飞地工业园建设、探索共同申报自由贸易区等方面开展合作。加快长平高速公路等重大项目建设，继续在科技、教育、卫生、社保、人才培训、干部交流等方面为平潭提供良好服务。进一步加强“三维”对接和专业化招商。深化与海内外重点客商以及异地商会、华侨华人社团的联系，积极开展涉侨引智引资引技工作，更加积极地维护侨益、凝聚侨心、发挥侨力，鼓励和支持海内外乡亲“回归”发展。实施技术、品牌、质量、服务带动出口战略，鼓励发展服务外包，积极推进外贸企业转型升级，着力开拓新兴市场。支持江阴国家汽车整车进口口岸发展壮大以及福州保税港区开展跨境电子商务零售出口试点。加大电子口岸建设力度，争取罗源湾港区扩大对外开放。进一步支持有条件的企业“走出去”或在境内外上市。全面拓展与港澳在金融、物流、旅游、文化创意等领域的合作。加强国际友城合作以及泛珠三角、闽浙赣皖、闽东北等区域协作、山海协作，继续抓好对口援疆援藏援宁工作。

　　全方位深化拓展榕台交流合作。加快台商投资区建设步伐，完善海峡两岸农业合作实验区、台湾农民创业园、闽台（福州)文化产业园等榕台产业合作平台。大力发展海峡旅游，扩大“两马”旅游品牌知名度和影响力，不断拓展赴台旅游客源市场。争取开通黄岐至马祖客运航线，进一步拓展榕台空中直航、海上直航。推动开展对台离岸金融业务，争取建立国家对台金融合作示范区。建设台胞协调服务平台，争创全国台胞权益保护工作先行先试示范点。深化榕台民间交流合作，争取榕台互设办事处，办好“5·18”海交会和一批特色鲜明、层次较高、影响力大的对台交流活动。

　　（二）加快培育壮大现代产业集群。

　　大力发展都市现代农业。认真实施“粮安工程”，稳定粮食种植面积，拓展引粮入榕渠道，加强粮食储备仓容建设，确保口粮绝对安全。积极发展设施农业、节水农业、循环农业、休闲观光农业，支持福清国家级现代农业示范区和长乐、闽侯、连江、闽清、罗源、永泰等现代农业示范基地发展。实施种业创新和产业化工程，着力培育一批“育繁推一体化”的种业企业和花卉苗木基地。推进福州茉莉花种植与茶文化系统申报全球重要农业文化遗产，建设闽侯金鱼产业园、福人花卉市场等一批特色农业项目。加快构建新型农业经营体系，进一步落实集体所有权、稳定农户承包权、放活土地经营权，培养造就新型农民队伍，大力扶持家庭农场、专业大户、农民合作社、产业化龙头企业等新型农业经营主体。完善农业支持保护体系、基层农业公共服务机构和农产品市场流通体系，建设市农业信息中心，发展农机、植保等专业化服务组织。

　　切实抓好工业园区提升和龙头项目建设。加快完善现有工业园区，推进数字福建产业园、中科数据产业园等新兴产业园区建设，打造各具特色与优势的产业集聚平台。创新工业园区建设管理体制，统筹产业布局和招商引资，强化产业配套协作。落实产业龙头促进计划，抓好神华煤港电一体化、马尾船政船舶与海洋工程装备园区特种船舶、申远新材料等133个项目建设，建成福清核电站1号机组、天辰耀隆己内酰胺等63个项目。

　　不断强化创新驱动与品牌带动。引导企业加大技改、研发投入，全面加强机械、纺织、食品、冶金、建陶、工艺品等传统产业的技术改造，争取新增省级以上创新型企业15家，新认定市级以上企业技术中心20家。加快发展新一代信息技术、高端装备制造、生物与新医药、新材料、新能源、节能环保等战略性新兴产业，力争高新技术产业产值突破3000亿元。积极开展产学研用协同创新，加强与知名高校、科研院所合作，支持建设院士（专家)工作站、博士后科研工作站和科技企业孵化器、知识产权维权援助中心等创新服务平台。认真实施闽都人才集聚工程，推进国际人才交流中心和人才公寓建设，建成国际人才项目孵化器、留学人员创业园（闽侯园)和大学生创业园，提高中国福州海西引智试验区建设水平。建立完善品牌评价、认定、拓展、保护和推动机制，支持企业并购国内外知名品牌，争创全国质量强市示范城市和全国知名品牌创建示范区。

　　持续提高服务业发展水平。扎实推进海峡金融商务区、闽江北岸中央商务区建设，认真抓好鼓楼区国家服务业综合改革试点，做大做强总部经济、楼宇经济。进一步提升海峡汽车文化广场及水产品、农副产品等批发交易市场，加快建设南通物流园等现代物流集聚区，大力发展第三方物流和冷链物流、城市配送物流。拓展信息消费，提速建设台江海峡电子商务产业基地二期等项目。积极构建海西现代金融中心，支持福建海峡银行、福州农商银行引进战略投资者，鼓励发展民营银行和小额贷款公司、村镇银行。打响闽都文化、温泉养生、休闲度假、自然生态等旅游品牌，推动“三坊七巷”创建国家5A级旅游景区和鼓岭创建国家级旅游度假区，推进华侨城、桂湖、贵安、东壁岛等一批文化旅游综合体项目建设。办好第16届中国连锁店展览会等大型展会。积极争取组建福州航空，鼓励航空公司增辟重点航线或在榕设立航空基地。

　　扎实推进“海上福州”建设。加快闽台（福州)蓝色经济产业园建设步伐，发展壮大临港工业、港口物流、造船、海洋运输、滨海旅游、海洋生物医药等“蓝色产业”。鼓励支持福州港资源整合、航线开辟，加大力度建设一批与福州港对接的陆地港，进一步拓展福州港经济腹地，提升福州港综合实力。稳妥有序推进罗源湾养殖退养，大力拓展湾外、境外养殖。支持发展远洋捕捞、水产品精深加工和水产品市场，推进连江现代渔业产业园和中国—东盟水产品交易所、恒兴南方水产品加工交易中心等建设。启动市级海洋功能区划修编，加强海域、海岸、海岛保护利用，建设洋屿岛海洋度假公园等项目。优化海域资源配置，开展游艇码头等经营性项目用海市场化配置工作试点。

　　（三）统筹推进新型城镇化和城乡一体发展。

　　加快实施福州新区重点区域开发。大力推进马尾新城三江口、闽江口等组团建设，加大琅岐开发力度。积极建设长乐航空新城，力争设立长乐空港综合保税区。依托环福清湾和江阴湾区域，科学规划建设福清海港新城。统筹罗源湾南北两岸基础配套和产业布局，打造罗源湾临港工业聚集区和综合物流集散中心。以产业发展、民生事业、基础设施等方面重大项目建设带动福州新区开发，抓好海峡文化艺术中心、滨海大通道、马尾大桥、城际轨道交通、东部快速通道、绕城高速公路东南段、道庆洲大桥等项目，建成或基本建成江阴港铁路支线、可门港铁路支线等项目，力争福州新区完成投资1000亿元。

　　切实提升中心城区功能品位。加强海峡奥体中心、火车南站、火车北站等片区配套设施建设、环境整治与管理提升，推进二环路、三环路、环南台岛滨江休闲路等道路沿线环境整治。综合整治200个老旧住宅小区，完成旧屋区改造280万平方米。加快光明港公园、飞凤山公园、江心公园等10个公园建设，实施西湖左海连通工程，新建环南台岛、左海公园至金牛山、金鸡山公园至温泉公园等城市绿道与慢行系统100公里。加大城区内河截污和南台岛内河整治力度，力争动工建设江北城区山洪防治及生态补水工程，健全完善内河长效管理机制。统筹推进城市灯光夜景工程建设。完善和提升城区路网等基础设施，争取新改扩建城市道路20公里、燃气管网20公里、排水污水管网29公里、市政供水管网44.5公里，基本建成福湾路、化工路、国货路等连接二环、三环的快速通道，改造金山大桥和紫阳立交、金鸡山隧道等一批道路交通节点。完成地铁1号线沿线站点土建工程，动工建设地铁2号线。积极推进“智慧福州”建设，实施智能公交系统、“数字城管”系统二期等项目。坚决治理违法建设、违规夜市摊点、非法采砂、渣土车违规、交通拥堵等突出问题，进一步提升城市管理水平。完成福莆宁同城化总体发展规划编制，加强交通建设、产业发展、公共服务等领域的同城化对接合作。

　　协调推动周边县（市)和小城镇建设发展。编制完成新型城镇化发展规划。继续实施规划、布局、建设、管理、政策等“五个统筹”机制，加快福清、长乐、闽侯、连江等县（市)融入福州中心城区。鼓励各县（市)因地制宜发展壮大特色产业，支持县（市)城关扩容提质、更新改造。大力开发建设龙祥岛，打造生态宜居岛。进一步推进省、市试点小城镇综合改革与建设，积极争取一批小城镇列入省级新型城镇化试点。科学制定差别化的户籍落户政策，放开建制镇和小城市落户限制，有序推进农业转移人口市民化。

　　努力改变农村面貌。不断加大强农惠农富农政策力度，推动公共财政投入向农村倾斜。加强农田水利基础设施建设，抓好土地整理复垦开发和在建的20个水利项目建设，解决20万农村人口饮水安全问题。认真执行村镇规划，严格农村住房建设管理。深入开展新一轮“百村竞赛”活动，强化农村环境综合整治，推进农村公路、户用沼气池、无害化卫生户厕等项目建设，打造一批“美丽乡村”精品示范村。加快健全农村留守儿童、留守妇女、留守老年人关爱服务体系。推动农村金融产品创新，建立适合农业农村特点的金融体系。深化扶贫开发和县（市)区对口协作机制，加大对财政困难县和老少边贫岛地区的帮扶力度，抓好整村推进扶贫开发、造福工程危房改造等工作，广泛开展“榕商联村”等活动。

　　（四）精心打造“美丽福州”。

　　推进节能减排攻坚。严把节能环保准入关，积极发展绿色经济、循环经济、低碳经济。实施50项重点节能项目、305项重点减排项目，加快建陶等行业清洁能源改造。开展生活垃圾分类和餐厨垃圾集中处置试点工作。完善环保基础设施，推进红庙岭垃圾焚烧发电厂二期等项目建设，建成洋里污水处理厂三期等项目。

　　加大环境保护力度。严格环保监管责任，完善环境监测体系。加强空气质量指数发布，实施区域大气联防联控，严控工业废气排放，确保空气质量持续位居全国前列。继续抓好闽江、敖江、龙江、大樟溪等重点流域水环境综合整治，加强畜禽养殖、石板材等重点行业污染治理和饮用水源区保护。加快构建水土资源流失治理和节约保护长效机制，造林绿化10.19万亩，进一步提升造林质量。坚决治理机动车尾气、垃圾焚烧、餐饮油烟、建筑施工扬尘和生活噪声等污染。强化重金属、危险废弃物、核与辐射环境监管，提高突发环境事件应急处置能力。

　　做好国家生态市创建工作。科学划定生态保护红线，落实主体功能区划，建立国土空间开发保护制度，加强资源环境承载能力监测预警。完善生态补偿和资源有偿使用制度，探索开展大樟溪北水南调水权交易试点，推进节能量、碳排放权、排污权等交易试点。严格执行生态环境损害赔偿制度，建立陆海统筹的生态系统保护修复制度。深入开展创建国家级生态县（市)区、生态乡镇等活动，倡导和推广绿色消费、“135低碳出行”等绿色生活方式。继续治理“青山挂白”，加强闽江河口湿地等重要湿地以及自然保护区、沿海防护林等重点生态功能区的保护。

　　（五）切实提高公共服务均等化水平。

　　办好人民满意教育。深化义务教育招生制度、“小片区”管理和农村薄弱校“委托管理”等改革。加大优质教育资源配置向农村尤其是边远、贫困、民族乡村倾斜力度，加强教师校际交流、农村支教和农民工子女入学等工作。推进学前教育普惠性发展和普通高中多样化、特色化建设，扩容公办幼儿园学位3000个和中小学学位5000个。提升职业教育办学水平，力争建成中等职业教育公共实训基地一期。鼓励和规范发展民办教育，扶持发展高等教育、特殊教育、老年教育、继续教育、社区教育。加强素质教育，建设中小学社会综合实践基地。进一步提高师德师风水平，培育名师群体。

　　深化健康城市建设。持续优化医疗资源布局，着手组建精神卫生等医疗联合体。深化公立医院改革，逐步建立财政补偿机制和医疗服务价格形成机制。鼓励社会资本参与公立医院改制重组，大力支持和规范社会资本办医。完善基本药物制度，推进医药流通体制改革。动工建设传染病院门诊综合楼、市一医院外科楼等项目，建成肺科医院负压病房楼、儿童医院病房楼等项目。加强基层尤其是农村医疗卫生人才队伍、医疗用房建设以及设备配备。提高传染病预警监测能力，强化重大疾病防控等工作。支持中医药事业发展，提升基层中医药服务能力。认真做好医患纠纷调处工作。深入开展爱国卫生运动。坚持计划生育基本国策，启动实施“单独两孩”政策，促进人口长期均衡发展。

　　繁荣发展文化事业。扎实抓好市属新闻媒体体制改革，加快已转制文化企业公司制、股份制改造。认真实施文化惠民工程，积极开展文化惠民“六进”等活动，提升一批乡镇综合文化站。争取建成海峡图书馆，设立一批城市街区24小时自助图书馆。支持文艺精品创作，繁荣发展哲学社会科学、文学艺术。大力弘扬闽都文化，加强历史文化名城保护和非物质文化遗产保护以及传统工艺美术传承创新，推进朱紫坊、上下杭历史文化街区，烟台山历史风貌区，船政文化遗址群等保护修复，建设海峡非物质文化遗产生态园。提高文化产业规模化、集约化、专业化水平，支持国家广告产业园、国家动漫影视实验园等项目建设与发展。

　　推动全民健身与青运同行。全力加快首届全国青年运动会场馆建设，建成海峡奥体中心“一场三馆”和运动员村。进一步提升竞技体育水平，积极做好第十五届省运会参赛和首届全国青年运动会备战工作。办好环福州·永泰国际公路自行车赛等活动，积累大型赛事办赛经验。组织开展全民健身活动200场以上，建设15个城市社区多功能运动场、200条健身路径，进一步打造中心城区和县（市)城关“十分钟”健身圈。

　　（六）积极开展社会治理创新。

　　强化社会服务管理。改进社会治理方式，推进政府治理和社会自我调节、居民自治良性互动。培育发展和规范管理社会组织，着手设立社会组织综合服务平台，积极稳妥开展社会组织直接登记。完善“大调解”工作体系和重大决策社会稳定风险评估、公共安全应急处置等机制，推行信访公开听证等制度，进一步加大信访积案化解力度，切实按照“路线图”依法处理信访事项。积极创建国家信息惠民示范城市，继续办好便民呼叫中心12345、政风行风热线等便民利民公共服务平台。加强流动人口、特殊人群服务和管理，抓好社区矫正、青少年事务社工等工作。支持工会、共青团、妇联等人民团体广泛参与社会治理及公共服务。加快少数民族地区发展，维护宗教领域和谐稳定。扎实做好防震减灾、地方志、科普、气象、保密等工作。

　　夯实社区建设等基础工作。深入开展全国和谐社区建设示范城市创建活动，提升网格化社会服务管理水平，加快城中村改制步伐，增强社区自治和服务功能。进一步减轻基层负担，完善社区、村干部选拔任用制度，建立健全居民、村民监督机制，推进村务公开、决策听证等基层民主建设。深化军民融合发展，加强国防教育、国防动员、国防后备力量建设和海防、人防、反走私等工作，实施科技拥军、“四个一好”等项目建设，巩固提升双拥模范城创建成果。

　　深化“平安福州”建设。完善立体化社会治安防控体系，强化治安源头管控，推进视频监控等系统建设，依法严密防范和惩治各类违法犯罪活动。切实抓好“六五”普法和法律援助、法律服务、司法鉴定、仲裁调解等工作。加强道路交通、建筑施工、校园、矿山等重点领域安全生产监管，完善隐患排查治理和安全预防控制体系，坚决防范和遏制重特大事故发生。健全防灾救灾体系，建成救灾物资储备库。

　　提升市民文明素质。继续推进以社会主义核心价值体系建设为引领的精神文明建设，加强社会公德、职业道德、家庭美德和个人品德教育。巩固文明城市建设成果，力争福清、长乐通过全国文明城市考评。强化未成年人思想道德建设，鼓励发展志愿服务，培育良好的社会风尚。加强政务诚信、商务诚信、社会诚信建设，大力建设“诚信福州”。

　　（七）着力多办惠民利民实事。

　　积极促进城乡居民收入持续稳定增长。深化收入分配制度改革，保护劳动所得，增加低收入者收入，扩大中等收入者比重，努力缩小城乡、区域、行业收入分配差距。健全工资决定和正常增长机制、劳动关系协调机制，完善最低工资、工资支付保障和企业工资集体协商制度。多渠道增加城乡居民财产性收入。完善城乡均等的公共就业创业服务体系，强化职业技能培训，促进以高校毕业生为重点的青年就业和农村转移劳动力、城镇困难人员、退役军人就业，争取城镇新增就业13.3万人，转移农业富余劳动力4.6万人。

　　持续提高城乡社会保障水平。稳步实施城乡居民社会养老保险一体化，适时提高城乡低保、农村五保标准和新农合年人均筹资水平，推进城乡居民基本医疗保险制度整合。完善社会养老服务体系，健全居家养老服务网络，发展老年服务产业，整合和新建一批老年活动场所。基本建成海峡妇女儿童活动中心。发展社会福利和社会救助，鼓励和规范慈善捐助，加快市社会福利中心、残疾人康复就业中心以及福乐家园建设。

　　认真解决群众关注的热点难点问题。加大交通综合整治力度，优化交通组织和管控，优先发展公共交通，新建一批立体停车场、地下停车场，新增更新公交车400辆、出租车600辆，新辟和优化公交线路20条，新建公共便民自行车站点100个。新开工建设7850套保障性住房，力争建成8000套保障性住房。大力推广“模拟征迁”等和谐征迁模式，完善对被征地、收海农民的保障机制。积极解决历史遗留“两权证”办理等问题。继续加强“菜篮子”工程建设，着力保障市场供应量足、质优、价稳。坚决治理“餐桌污染”，抓紧建立健全农产品质量和食品安全追溯体系，严厉打击食品药品违法犯罪。

　　（八）大力建设法治政府和服务型政府。

　　加快转变政府职能。深入推进政企、政资、政事、政社分开，全面完成新一轮政府机构改革，积极稳妥开展事业单位分类改革。进一步加大简政放权力度，切实把该放的放下放彻底，该管的管好管到位。积极试行政府投资项目审批代办制，开展重大项目审批“直通车”服务。建成市民服务中心，加快各级行政（便民)服务中心（代办点)标准化建设。

　　全面推进依法行政。坚持用制度管权管事管人，完善政府信息公开、重大事项听证等制度，严格依照法定权限和程序行使权力、履行职责。依法接受人大及其常委会的法律监督、工作监督，主动接受政协的民主监督，自觉向人大报告、与政协协商政府重大事项和重大决策，广泛听取各民主党派、工商联和各人民团体、社会各界人士的意见。进一步提高政府立法和办理人大议案、政协提案的质量。认真落实行政执法责任制，加强行政复议、行政诉讼等工作。

　　始终坚持为民务实。认真开展党的群众路线教育实践和“四个万家”等活动，持之以恒落实中央“八项规定”精神，坚决整治形式主义、官僚主义、享乐主义和奢靡之风，更加及时有效地为基层、企业和群众解决实际问题。自觉把群众呼声作为第一信号、群众利益作为第一选择、群众满意作为第一标准，集中财力多办打基础、利长远、惠民生的实事好事。健全完善惩治和预防腐败体系，强化行政监察和审计监督，扎实推进廉政建设和反腐败斗争，确保人民赋予的权力始终用来为人民谋利益。

　　切实做到“马上就办”。自觉以“踏石留印、抓铁有痕”的劲头推进工作，立说立行、真抓实干，进一步增强政府的执行力和公信力。大力倡导敢于负责、敢于担当、敢闯敢试的精神，不断提高攻坚克难、推进发展的能力与水平。深入开展绩效管理工作，严格效能问责和岗位绩效考核，坚决反对敷衍塞责、推诿扯皮，用铁的纪律坚决治理“庸懒散”等问题。

　　各位代表，全面深化改革的征程已经开启，前进的号角催人奋进。让我们紧密地团结在以习近平同志为核心的党中央周围，在省委、省政府和市委的坚强领导下，进一步敢为、能为、有为，为推进福州新区开放开发，在更高起点上加快建设闽江口金三角经济圈，率先全面建成小康社会而努力奋斗！
